# Are pregnancy outcomes affected by the lack of legal status? A demographic study based on 850,288 live births in Switzerland

**DOI:** 10.1186/s12884-023-05870-5

**Published:** 2023-08-05

**Authors:** Caterina Montagnoli, Philippe Wanner

**Affiliations:** 1 Institute of Demography and Socioeconomics, University of Geneva, Geneva, Switzerland; 2 Midwifery Degree Programme, Geneva School of Health Sciences, HES-SO University of Applied Sciences and Arts of Western Switzerland, Geneva, Switzerland; 3 Institute for Biomedical Ethics, University of Basel, Basel, Switzerland

**Keywords:** Health care disparities (MeSH), Irregular migrants, Maternal-child Health Services (MeSH), Preterm birth, Low and very low birth weight, Small for gestational age, Switzerland (MeSH)

## Abstract

**Background:**

In the context of increased global mobility, it is fundamental to understand migrants’ needs and how governments can ensure equal health opportunities for both regular and irregular migrants simply by applying low-cost primary health care measures. To identify health issues in which to intervene, this study analysed the impact of a mother’s lack of legal status, together with available biological and socioeconomic characteristics, on four indicators of adverse perinatal outcomes in Switzerland.

**Methods:**

Based on the exhaustive records of the Swiss Federal Statistical Office (FSO) for its Vital Statistics (BEVNAT), different indicators of birth outcomes, including preterm birth (PTB), low and very low birth weight (LBW and VLBW), and small for gestational age (SGA), were analysed using logistic regressions on live births occurring from 2005 to 2018. These four adverse outcomes were defined as dependent variables. Statistical analysis was performed using the statistical package STATA, version 17.

**Results:**

Selected pregnancy outcomes were conversely affected by an irregular legal status. Analysis run on the final sample showed that, compared to the neonates of mothers who are non-migrant legal residents in Switzerland, newborns of irregular migrants have higher risks of PTB (aOR 1.18 95% CI [1.05-1.32], *p*<0.01) and VLBW (aOR 1.43 [1.13-1.81], *p* < 0.01]). In contrast, we observed that in both irregular and regular migrant groups, the odds of SGA were lowered (aOR .76 [.68-.85] *p*<0.01) and aOR .93 [.91-.94], *p*< 0.01, respectively). A similar effect was observed when controlling for any adverse outcome (any AOs) (aOR  .90 [.83-.99] p 0.022; and aOR .93 [.91-.94] *p*< 0.01, respectively).

**Conclusions:**

Our results, together with those from the available literature, call for a more comprehensive assessment of all pregnancy outcomes as well as of the social determinants of health for which the analysis was adjusted. Given the complexity of the migration phenomenon, future studies should account for local structural restrictions in the organization of care, the extension of a person’s network as a means of health care accessibility, diverse backgrounds and cultures and the recent arrival status of migrants. This would allow researchers to understand the long-term impact of social determinants of health on the wellbeing of a mother and child and take them into account in the adoption of health policies.

**Supplementary Information:**

The online version contains supplementary material available at 10.1186/s12884-023-05870-5.

## Background

Like many European countries, Switzerland has a growing migrant population, fuelled by the presence of numerous international organizations and multinational organizations, its central position in Western Europe, a labour market in expansion and its important economic growth [1].

Due to a restrictive naturalization law, the naturalization rate of foreign citizens in Switzerland is among the lowest in Europe (1–2%), and its requirements and processes are very difficult to meet [1]. As a foreigner, passing through a three-tiered (communal, canton and federal) decision system is the only way to obtain the Swiss citizenship together with proving at least 10 years of continuous residency in the country. Instead of the *ius soli*, or “the principle that the nationality of a person is determined on the basis of their country of birth” [2], Switzerland applies otherwise the so called *ius sanguinis* where citizenship is transmitted through paternal or maternal descent. Accordingly, having foreign nationality can be used as a proxy for migrant status. Assessments from the 2020 Swiss Migration Report revealed that the foreign resident population included 2,151,854 people, of which 680,909 were non-European Union (EU) nationals [3, 4]. These numbers, which make up 24% and 7.8% of the total Swiss population respectively [5], have constantly grown since the last century. Among regular residents, Germany, France, Italy, and Portugal are the four EU countries of origin that are the most represented, whereas Turkey, Kosovo and North Macedonia are the non-EU countries that are the most represented [6].

In addition to the migrant population with a valid residency permit, a number projected to be between 80,000 and 100,000 migrants living in Switzerland do not have a valid permit [7]. They are called the *sans-papiers*, literally meaning ‘without papers,’ or irregular migrants, defined as “people in the global context who—owing to irregular entry, a breach of a condition of entry or the expiry of their legal basis for entering and residing—lack legal status in a transit or host country” [8]. Official data concerning this population are rarely available, yet a national investigation conducted in 2015, which involved 61 contacts including migration dedicated services, NGOs and welcoming centres in Switzerland, projected that the largest migrant ethnic group among sans-papiers is represented by Latin-Americans followed by people from non-European countries, Africa and South-East Asia [9].

According to official data, in Switzerland, the migration movement is almost equally represented by the two sexes, with a small majority of women (51% women vs. 49% men) [10]. As of 2020, official data stated that most incomers belonged to the age group of 35–39 years, followed by the age groups of 30–34 years and 40–44 years [4]. These data are in line with the current literature that affirms that women and men of reproductive age are more willing to migrate for new working opportunities [11].

Due to the important presence of migrants in Switzerland, the social and structural integration of this group is an important issue to ensure equal opportunities regardless of the country of origin. Accordingly, both the Swiss government and the OECD have agreed on different measures that have been proven to determine the degree of integration of migrants into the receiving society [12, 13]. The goal of receiving countries adopting tailored integration policies is to increase social participation and contribution [14] and to measure this phenomenon, the OECD has suggested, among others, *self-reported medical status* and *the degree of unmet medical needs* as indicators of integration [13]. Similarly, the Swiss government has outlined 68 items reflecting integration levels, including “access to health care” and “the infant mortality rate” [12].

As we move towards a more diverse population with different health needs, it seems fundamental to be aware of their characteristics and how governments could boost the integration of both regular and irregular migrants by simply applying low-cost primary health care measures [15].

To provide an understanding of the effect of integration policies on the health of migrant populations of reproductive age, this study calculated the effect of an irregular status in Switzerland on pregnancy outcomes. The current literature systematically refers to four specific outcomes as consequences of availability, accessibility and compliance with health care during pregnancy and long-term health after birth, especially in irregular migrant populations [16–18]. These include preterm birth (PTB), low and very low birth weight (LBW and VLBW) and small for gestational age (SGA).

This study presents an analysis of these perinatal health indicators from a Swiss Federal Statistical Office database providing micro data on livebirths between 2000 and 2018.

## Methods

The Swiss Federal Statistical Office provided anonymous data on births occurring in Switzerland between 2000 and 2018 from their registration system, including both women with Swiss and foreign nationality and both women legally living in Switzerland or abroad. The dataset included information about the year and the day of birth, the sex of the baby, maternal parity, the nationality of the newborn and that of the mother, the mother’s municipality of residence (either in Switzerland or abroad), the mother’s civil status, the office of registration of the birth, the civil registration number, the paternal residence status and, of course, the gestational age and weight of the newborn at birth. This dataset included 1,675,062 live births between 2000 and 2018 in Switzerland. Due to a critical change in data registration in April 2004, which included, among others, gestational age at birth and maternal foreign domicile, we excluded births occurring prior to 2005, leaving 1,292,225 entries for analysis. After further verification, we noticed that in the dataset gestational age entries were incomplete until late-2006. In particular, the totality of all 67,076 gestational age information were missing in 2005 as well as 63,629 in 2006 for a total of 185 births’ gestational age information.

Irregular migrants were identified as people with foreign residence who gave birth in Switzerland with the exclusion of medical tourists or frontier workers. As Switzerland is well known for its medical tourism, non-resident women with certain nationalities were identified as tourists. These included women from Saudi Arabia, Russia, the United Arab Emirates, the United States of America, the United Kingdom and Spain [19]. No Canadian women gave birth in Switzerland in the selected time frame. After data validation, we added people from Israel to the medical tourist group. The frontier workers category included people from the 5 neighbouring countries, namely: Italy, France, Germany, Austria, and the Principality of Liechtenstein. A fourth category was added that included foreigners with an EU/European Free Trade Association (EFTA) residence who gave birth in Switzerland. Irregular migrants were then defined as foreigners with a legal residence in a non-UE/EFTA country that is not affected by medical tourism. To compare residents and irregular migrants only, we excluded births of medical tourists, EFTA residents and frontier workers from the analysis. After clearing the dataset from the tourist category as well as the category of frontier workers and EU nationals, we ended up with 1,022,501 data entries to analyse.

To verify that the number of births from the irregular migrants category was compatible with the estimated data, we calculated, based on previous research [15], the estimated number of births per year in the Geneva canton, and we compared it to the other cantons that welcome the highest number of irregular migrants (Zurich, Vaud, Basel and Bern) [9]. The results were comparable to those expected.

Four short-term adverse perinatal outcomes were studied: low birth weight (LBW) and very low birth weight (VLBW), including a weight at birth lower than 2500 g or 1500 g, respectively, preterm birth (PTB), including births before the 37th week of pregnancy, and small for gestational age (SGA), including birthweights inferior to the 10th percentile at a given gestational age. We also studied the odds of occurrence for at least one of these outcomes. We calculated the reference terms of SGA by looking at the mean weight, the standard deviation (SD) and the 10th percentile in each week after week 24 and until week 43. We then compared Swiss data with US data to confirm the compatibility of our results [20]. Further variable clearing, which included removing extreme recorded cases such as babies born prior to 24 weeks or in the 43rd week as well as babies born weighing less than 500 g or over 5500 g, removed an additional 1,610 entries from the data analysis. This allowed our analysis to be compared to those of the general population.

In the Supplementary Materials Document, we provide four tables showing the results computed with the whole dataset and the Swiss residents’ data as well as one figure showing the data clearing process.

Logistic regressions were run to measure the association of risk factors, namely, a lack of legal status, parity, maternal civil status, and the country of provenience or canton of birth, with the different outcomes. Civil status and canton of birth were then considered social determinants of health according to the WHO’s definition [21]. Literature reports poorer health among unmarried women [22]. The intrinsic organisation of the Swiss healthcare system, where the federal rules are adapted to cantonal needs and available services to the general or specific public, allowed us to consider the variable “canton of birth” as another social determinant of health [23]. Finally, in line with groundwork literature, we also considered the lack of legal status a social determinant of health [24].

Logistic regressions were run with two different models. The first reflected the legal status of the mothers (irregular or regular migrants), while the second focused on the origin of irregular migrant mothers. Because of the gestational age missing values, we ran logistic regressions with 993,856 data entries for the outcomes concerning birth weights and 850,411 for those concerning gestational age. Logistic regressions provide the probability (p) of one perinatal outcome according to the dimensions under study and the control variables (e.g., parity or civil status). For all models, the levels of significance (*p* < 0.05; *p* < 0.01) are presented to facilitate the interpretation of the results. Statistical analysis was performed using the statistical package STATA, version 17 [25].

## Results

### Descriptive results

After clearing the dataset, the distribution of livebirths per year with respect to the status of residence of the mother was presented in Table 1. The total number of births among legal or irregular residents in Switzerland slightly increased during the period under study, from 67,036 to 77,359 births. The number of births among irregular migrants progressively increased from 2005 to 2018, from 1,296 to 2,194 births. In Table 1, we also presented the row percentages of the Irregular migrants’ continents of origin. Overall, Latin American was the most represented with 31.36% of all irregular migrant births occurring in mothers of Latin American origin, compared to 17.64% among Asian mothers. For almost one-third (29.2%) of the irregular migrants, the nationality was unknown.

**Table 1:** Livebirths per year and maternal residency status

	Residency							
	Swiss residents	Regular migrants	Irregular migrants	Extra EU/EFTA	Africa	Latin America	Asia	Unknown
**Year**								
2005	46,608	19,165	1,296					
Row %	69.49	28.58	1.93	8.50	13.20	51.29	17.15	9.86
2006	47,056	19,053	1,701					
Row %	69.39	28.10	2.51	7.14	13.64	43.83	21.32	14.07
2007	47,635	19,154	1,915					
Row %	69.33	27.88	2.79	9.29	13.34	46.74	18.38	12.06
2008	48,891	19,338	1,884					
Row %	69.73	27.58	2.69	5.48	10.14	33.94	15.69	34.69
2009	49,759	19,410	1,979					
Row %	69.94	27.28	2.78	5.83	8.82	28.51	14.48	42.31
2010	50,528	20,072	1,873					
Row %	69.72	27.70	2.58	6.21	11.68	31.24	15.42	35.38
2011	50,321	19,960	2,028					
Row %	69.59	27.60	2.80	11.16	12.21	30.93	17.40	28.23
2012	50,760	20,675	2,034					
Row %	69.09	28.14	2.77	9.97	10.79	28.11	18.13	33.00
2013	50,645	21,118	2,104					
Row %	68.56	28.59	2.85	9.60	12.27	27.17	17.28	33.68
2014	51,552	21,655	2,153					
Row %	68.41	28.74	2.86	10.20	12.53	26.54	18.37	32.31
2015	52,119	22,201	2,255					
Row %	68.06	28.99	2.94	10.92	12.40	28.57	18.89	29.10
2016	52,237	23,167	2,223					
Row %	67.29	29.84	2.86	10.47	16.03	27.89	16.58	29.04
2017	51,911	22,998	2,116					
Row %	67.40	29.86	2.75	12.12	15.29	27.54	20.07	24.98
2018	52,490	22,668	2,194					
Row %	67.86	29.30	2.84	12.55	16.19	30.23	19.70	21.32
Total	702,512	290,634	27,755					
Row %	68.81	28.47	2.72	9.33	12.65	31.36	17.64	28.97

*EU *European Union, *EFTA *European Free Trade Association. Source: Federal Statistical Office (FSO) for its Vital Statistics (BEVNAT). After the exclusion of births with extreme or missing values for birthweight and/or gestational age

In Table 2, we compared parity, civil status, and canton of childbirth with the residency status of the mother. Here, we noticed that irregular migrants had overall smaller families compared to their regular counterparts and Swiss residents (55.4% vs. 50.3% and 47.0%, respectively, for primiparas), with primiparous women having the most births. Nevertheless, in proportion, irregular migrants also presented the largest proportions of having four or more children (4.1 vs. 3.1 vs. 3.7%, respectively).

**Table 2:** Maternal characteristics per residency status

	Irregular migrants, N (col %)	Residency, N (col %)		Irregular migrants’ origins (col %)				
		Swiss residents	Regular migrants	Extra EU/EFTA	Africa	Latin America	Asia	Unknown
**Parity**								
One	15,384 (55.43)	330,160 (47)	146,215 (50.31)	67.71	60.57	60.94	64.67	64.16
Two	8,457 (30.47)	261,254 (37.19)	102,796 (35.37)	24.24	27.95	29.56	25.56	24.46
Three	2,784 (10.03)	85,070 (12.11)	32,005 (11.01)	6.34	8.45	7.77	7.10	7.65
4 + children	1,130 (4.07)	26,028 (3.71)	9,618 (3.31)	1.70	3.03	1.74	2.68	3.72
**Maternal civil status**								
Married	19,859 (71.55)	552,722 (78.68)	245,394 (84.43)	51.68	68.32	64.90	75.40	29.34
Not married	7,896 (28.45)	149,790 (21.32)	45,240 (15.57)	48.32	31.68	35.10	24.60	70.66
**Canton of childbirth**								
Geneva	3,351 (12.07)	33,565 (4.78)	27,051 (9.31)	23.76	16.86	20.16	13.41	3.00
Vaud	2,225 (8.02)	67,822 (9.65)	38,542 (13.26)	13.91	11.83	8.56	7.55	14.39
Zurich	4,105 (14.79)	130,918 (18.64)	58,500 (20.13)	14.32	12.50	10.84	14.96	16.54
Basel	953 (3.43)	26,669 (3.8)	12,084 (4.16)	4.05	3.03	2.96	3.41	1.29
Bern	3,748 (13.50)	103,519 (14.74)	27,495 (9.46)	8.26	11.12	10.35	12.12	25.48
Other cantons	13,373 (48.18)	340,019 (48.4)	126,962 (43.68)	35.70	44.65	47.13	48.55	39.30

Source: Federal Statistical Office (FSO) for its Vital Statistics (BEVNAT). *EU *European Union, *EFTA *European Free Trade Association, *CH *Switzerland, *col % *column percentage, *N *Number

Regarding civil status, irregular migrants also had the highest proportions of unmarried women giving birth in Switzerland compared to their regular counterparts and Swiss residents (28.45% vs. 15.57% and 21.32%, respectively). These numbers reflected the extensive regular inflow represented by high-skilled officers moving with their families to Switzerland for business purposes.

Among the five largest receiving cantons that welcome more than half of the births of irregular migrants, Zurich, Bern and Geneva represented the top three. The proportions of childbirths from irregular migrants in these cantons were 14.79%, 13.50%, and 12.07% respectively. While Geneva had the largest proportions of European citizens from non-EU/EFTA countries, Latin American irregular migrants and African irregular migrants, Zurich had the largest proportion of Asian irregular migrants.

We then computed the SGA limits, or the 10th percentile of weight for gestational age at birth, both for all livebirths and for the births of Swiss residents only. International standards available in the literature frequently refer to US data [20]. Yet, North American and European behavioural lifestyles, like eating habits and commuting methods, greatly differ as well as their overall accessibility of their respective health care systems. These differences inspired us to compute our specific Swiss SGA parameters. One reason why we chose to separately calculate the SGA births of the general birthing population and Swiss residents is to document the specificities of irregular migrants. A recent study from the Canadian statistical office analysing the unforeseen increase in SGA births between 2000 and 2016 has in fact supported how the demography of childbirth, or the change in the population composition over the 16-year period, partially explained the increasing SGA trend, suggesting the need for culturally sensitive curves [26]. In our case, however, this trend was not confirmed, and differences between Swiss residents and the general birthing population were minimal (see tables in the Supplementary Materials Document).

### Logistic regression results

After categorizing the data for the available variables, we performed logistic regressions for each of the four outcomes considered with and without controlling for parity, civil status and the canton where the birth was registered. We excluded tourists, EU/EFTA residents and the frontier population to directly compare irregular migrants with two groups: regular residents, being Swiss or migrants (Model 1) and migrants only (Model 2).

For unadjusted analysis, which can be found in Table 3 of the Supplementary materials document, logistic regressions showed significantly increased odds among irregular migrants for all included outcomes except for SGA. The results were similar when comparing irregular migrants with Swiss residents (Model 1) and with regular migrants (Model 2). In contrast, associations between a lack of maternal legal status and the odds of SGA were not significant (*p* = 0.830 and *p* = 0.321, respectively).

**Table 3:** Controlled logistic regressions between newborn outcomes and the independent social variables for the Swiss population, regular migrants, and irregular migrants

	SGA (*n* = 850,411)			PTB (*n* = 850,411)			LBW (*n* = 993,856)			VLBW (*n* = 993,856)			Any AO (*n* = 850,288)		
	aOR	[95% CI]	p	aOR	[95% CI]	p	aOR	[95% CI]	p	aOR	[95% CI]	p	aOR	[95% CI]	p
**Residency**															
Swiss resident	1 (ref.)			1			1			1			1		
Irregular migrant	0.76	0.68-0.85	0.0	1.18	1.05–1.32	0.004	0.95	0.85-1.06	0.375	1.43	1.13–1.81	0.003	0.90	0.83-0.99	0.022
Regular migrant	0.93	0.91-0.94	0.0	0.93	0.92-0.95	0	0.92	0.91-0.94	0.0	1.08	1.04–1.14	0.00	0.92	0.91-0.94	0.0
**Parity**															
One	1 (ref.)			1			1			1			11		
Two	0.60	0.59-0.61	0	0.90	0.88-0.91	0	0.82	0.80-0.83	0	0.88	0.84-0.92	0	0.68	0.67-0.69	0
Three	0.54	0.52-0.55	0	1.05	1.02–1.08	0	0.93	0.91-0.95	0	1.02	0.95-1.09	0.653	0.70	0.68-0.71	0
4 + children	0.53	0.51-0.56	0	1.34	1.28–1.39	0	1.10	1.05–1.15	0	1.20	1.08–1.34	0.001	0.80	0.78-0.83	0
**Maternal civil status**															
Married	1(ref.)			1			1			1			1		
Not married	1.23	1.21–1.25	0	1.10	1.08–1.12	0	1.23	1.21–1.26	0	1.42	1.36–1.50	0	1.19	1.18–1.21	0
**Canton of childbirth**															
Other cantons	1 (ref.)			1			1			1			1		
Geneva	1.08	1.05–1.11	0	1.33	1.29–1.38	0	1.45	1.41–1.50	0	1.86	1.72–2.03	0	1.19	1.16–1.22	0
Vaud	1.21	1.19–1.24	0	1.24	1.21–1.28	0	1.37	1.34–1.41	0	1.96	1.84–2.10	0	1.22	1.20–1.24	0
Zurich	0.92	0.90-0.93	0	1.11	1.09–1.14	0	1.11	1.09–1.14	0	1.52	1.43–1.61	0	0.99	0.97 − 1.00	0.139
Basel	0.98	0.94-1.02	0.248	1.36	1.31–1.42	0	1.40	1.35–1.46	0	2.68	2.46–2.93	0	1.12	1.09–1.16	0
Bern	1.03	1.01–1.05	0.010	1.18	1.15–1.21	0	1.24	1.21–1.27	0	2.03	1.90–2.15	0	1.09	1.07–1.11	0

Source: Federal Statistical Office (FSO) for its Vital Statistics (BEVNAT). *AO *Adverse Outcome, *SGA* Small for Gestational Age, *LBW *Low Birth Weight, *VLBW *Very Low Birth Weight, *PTB *Preterm Birth, *EU *European Union, *EFTA *European Free Trade Association, *CH *Switzerland

Note: the models were run differently according to the availability of the data

The results of our adjusted logistic regressions comparing irregular migrants with the entire legal population are shown in Tables 3 and Table 4. Compared to the neonates of mothers who were legal residents in Switzerland, those born to irregular migrants had higher risks of PTB (aOR 1.18 [1.05–1.32], *p* < 0.01) and VLBW (aOR 11.43 [1.13–1.81] *p* < 0.01) than Swiss natives. In contrast, regular migrants had lower odds of PTB (aOR 0.93 [0.92-0.95], *p* < 0.001). When looking at the region of origin of irregular migrants, as shown in Table 4, we noticed that the ones from the extra EU/EFTA countries had higher odds for PTB (aOR 1.39[1.12–1.72] p 0.003) and VLBW (aOR 1.60 [1.01–2.53] p 0.044) but lower risks for SGA (aOR 0.67 [0.54-0.86] p 0.001). Latin American origins are protective against SGA (aOR 0.65 [0.53-0.79] p 0.0); LBW (aOR 0.77[0.64-0.93] p 0.007) and Any AO (aOR 0.82 [0.71-0.95] p 0.009).

**Table 4:** Controlled logistic regressions between newborn outcomes and the independent social variables for irregular migrants by maternal origin

	SGA (*n* = 850,411)			PTB (*n*= 850,411)			LBW (*n* = 993,856)			VLBW (*n* = 993,856)			Any AOs (*n* = 850,288)		
	aOR	[95% CI]	p	aOR	[95% CI]	p	aOR	[95% CI]	p	aOR	[95% CI]	p	aOR	[95% CI]	p
**Maternal origin**															
Extra EU/EFTA	0.67	0.54-0.86	0.001	1.39	1.12–1.72	0.003	1.09	0.88 − 1.40	0.430	1.60	1.01–2.53	0.044	0.95	0.80-1.12	0.542
Africa	1.00	0.76-1.32	0.994	1.23	0.90-1.68	0.194	1.17	0.89-1.53	0.270	2.00	1.17–3.41	0.011	1.06	0.84-1.32	0.632
Latin America	0.65	0.53-0.79	0.000	1.09	0.89-1.32	0.404	0.77	0.64-0.93	0.007	1.03	0.68-1.56	0.891	0.82	0.71-0.95	0.009
Asia	0.70	0.50-0.99	0.041	1.07	0.76 − 1.50	0.715	0.94	0.68 − 1.30	0.701	1.35	0.67-2.72	0.406	0.83	0.64-1.07	0.153
Unknown	0.93	0.85-1.01	0.078	0.81	0.73-0.90	0.00	0.88	0.79-0.98	0.016	1.14	0.90-1.44	0.266	0.90	0.83-0.96	0.002

Source: Federal Statistical Office (FSO) for its Vital Statistics (BEVNAT). *AO *Adverse Outcome, *SGA* Small for Gestational Age, *LBW *Low Birth Weight, *VLBW *Very Low Birth Weight, *PTB *Preterm Birth, *EU *European Union, *EFTA *European Free Trade Association, *CH *Switzerland

Note: the models were run differently according to the availability of the data

In contrast, as shown in Table 3, we observed that people without regular permits did not have higher odds of SGA: when controlling for parity, married civil status and canton of childbirth; the data showed a lower ratio of this adverse outcome in both irregular and regular migrant groups (aOR 0.76 [0.68-0.85], *p* < 0.01; aOR 0.93 [0.91-0.94], *p* < 0.01, respectively). A similar effect was observed when controlling for any adverse outcome (any AO) with the available social variables (aOR 0.90 [0.83-0.99], p 0.022; aOR 0.92 [0.91-0.94], *p* < 0.01, respectively). While a significant association was accounted for by regular migrant status and lower odds of LBW (aOR 0.92 [0.91-0.94], *p* < 0.01), we did not find any significant association between LBW and the absence of legal status (*p* = 0.375).

Interestingly, higher risks of SGA were revealed when childbirth occurred in the cantons of Geneva, Bern or Vaud, irrespective of the legal status of the birthing person.

 Associations between the included adverse outcomes and irregular status among the migrant population are presented in Table 4 of the FSupplementary Materials Document. The results were similar to those presented for irregular or regular migrants vs. the Swiss population, with significant associations and increased odds for PTB and VLBW, significant associations and decreased odds for SGA and any adverse outcome and with no association for LBW in the migrant population.

## Discussion

In this study, we used the Swiss Federal Statistical Office (FSO) for its Vital Statistics (BEVNAT) database to discuss whether a lack of legal status adversely affects preterm birth (PTB), low and very low birth weight (LBW and VLBW), small for gestational age (SGA) or any of these four outcomes. The analyses showed that, after controlling for the available socioeconomic and biological factors, the risks for PTB and VLBW were higher for women without a regular permit. No significant difference was found for LBW neonates, while irregular migration was associated with a lower risk of SGA or any of the adverse outcomes considered. While these results seem inconsistent with each other, when analysing the literature, we found various similarities, which are explained in the following paragraphs.

Recent research has focused on the impact of timing and adequacy of antenatal care on pregnancy outcomes for irregular migrants across Europe. While their included populations were small and time restricted compared to ours, similar outcomes emerged when analysing short-term pregnancy outcomes. In a Danish cross-sectional study published in 2021, no major pregnancy complications were measured among irregular migrants [27]. Similarly, no higher rates of PTB, LBW or still births were accounted for in the Irregular population of a Finnish cross-sectional paper published in 2021 [17]. Going back slightly further, Belgian observational and retrospective studies published in 2020 also found no significant differences in obstetrical outcomes between regular and irregular populations [18, 28]. Older Swiss analyses (2008) has also reported results comparable with those from northern Europe [29].

In contrast with these findings, a large Swedish cohort study published in 2019 found that there was actually an increased risk for PTB and LBW for their included population of refugee or irregular women [30]. Similarly, a Dutch cohort study in 2011 also found increased odds of PTB and LBW among Irregular vs. documented mothers [31].

The lack of convergence of the available literature, together with our results, calls for a more comprehensive assessment of pregnancy outcomes as well as of the social determinants of health for which the models were adjusted.

Relevant literature has proved that equal health opportunities for all migrants could be improved by simply introducing accessible primary health care measures [15, 32]. In Switzerland, women not holding a regular permit might still benefit from basic medical care thanks to the 1996 law on health care insurance [33]. According to Schmidt and colleagues [34], all irregular migrants are subject to structural restrictions in the organization of care, which is often fragmented and decentralized because of time and working duties, and to disruptions in the provision of care. Not to mention the legal restrictions which pose a major access barrier for irregular migrants’ entitlements and rights to health [35]. One would therefore expect less favourable perinatal outcomes. The type of services provided to women was not included in our analysis, and this could have affected the final results for those who encountered obstacles during pregnancy. Nevertheless, we know that each cantonal healthcare system has its own internal organisation for what concerns healthcare accessibility for irregular migrants. Our results show consequences connected to the diverse arrangements over the country only for SGA in the canton of Zurich (aOR.92[0.90-0.93] *p* < 0.01). Yet, data detailing whether the person benefitted a specific service were not available thus a conclusion cannot be drawn.

It is also arguable that considered pregnancy outcomes would only reflect short-term health and not the long-term impact of social determinants of health on the wellbeing of a mother and growing newborn. It has been globally acknowledged that long-lasting health builds on not only the 9 months of pregnancy but also from conception until the second year of age, the window known as the first 1,000 days [36–39]. In this sense, newborns’ outcomes, as a result of maternal health-status during pregnancy and as the underpinning foundations for future long-lasting child health, should be considered together with maternal and neonatal relevant social determinants of health.

Another important aspect that emerged in a Swiss prospective study is the extension of a person’s network as a means of health care accessibility [40]. The greater the network, the better the person understands dedicated health care services and their uses. Further assumptions project that recently arrived migrants still benefit from the so-called healthy migrant effect, which has been proven to play a role in perinatal outcomes [41].

The diverse backgrounds and cultures of irregular migrant populations may also play a role in maintaining good health during pregnancy irrespective of their adequacy of antenatal care attendance, as suggested in the birth weight paradox model or the “Latina paradox” [42]. After consideration and adjustment of our analysis for maternal countries of origin, findings have shown that the absence of a legal permit may act as a protective factor for mothers with Latin American origins for most outcomes (SGA: aOR 0.65 [0.53-0.79] *p* < 0.01 ; LBW: aOR 0.77 [0.64-0.93] p 0.007; any AOs: aOR 0.82 [0.71-0.95] p 0.009). Previous studies analysing data from the European and north American receiving countries, repeatedly and consistently reported a “healthy migrant effect” on the birth outcomes of migrant women from lower socioeconomic status when compared to their local counterparts [43, 44]. Recent literature has also pointed out that the extension of migrants’ network might play a crucial role in their (perceived) health [40]. In our case, we believe that the extension of this community across the country—especially in Geneva with its international population—together their well-established internal networks, provide protection against negative outcomes creating the foundation of the Swiss version of the Latina Paradox. The diversity of the migrant population together with the geographical position of the biggest maternities could also explain why SGA odds were higher in the Geneva, Bern and Vaud cantons irrespective of the migration status.

## Strengths and limitations

In analysing the changes in four pregnancy outcomes in the Swiss population, this is, to our knowledge, the most recent study addressing pregnancy outcomes among the irregular migrant population. Nevertheless, given the complexity of the migration phenomenon incomplete available data remain a limitation.

The measurement of the impact of socioeconomic and migratory factors on the risk related to pregnancy and childbirth is limited by the availability of data. First, the dataset had to be extensively revised before being analysed: due to the new registration methods, all data entries between 2000 and 2004 were excluded from the analysis, and extreme cases of gestational age (< 24 or > 43 weeks) and birthweight (< 500 or > 5,500 gr) were cleared. This might have led to a potential population selection bias where only women delivering since 2005 or 2007 were included. Moreover, the definition of irregular migrants is based on the place of residence and citizenship. Finally, no precise information of the status of residence of the women was available (short-term visits in Switzerland, long-term residence as irregular workers, etc.).

As previously mentioned, our analysis did not distinguish irregular migrants with health care insurance from those who were not able to benefit from it. Starting from 2002, irregular migrants must pay for their health care insurance irrespective of their legal status [33]. Next, critical measures such as maternal age or multiple births, which might have affected or better explain our results, were not provided within the dataset. Finally, comorbidities and other population-related or medical factors that could have impacted the four pregnancy outcomes were not recorded and thus were not considered.

## Conclusions

Migration is expected to remain a characteristic feature of worldwide population. Switzerland, with its dynamic job market, will undoubtedly stay one of the largest magnets of attraction for migrants. To facilitate their integration, a thorough understanding of this population’s characteristics should be pursued.

The results of the current demographic analysis, together with those of the literature, suggest that the current knowledge on irregular migrants’ health outcomes are not comprehensive of some key aspects of life, such as social determinants of health, that are likely to impact one’s health.

Literature based hints on these facets suggest that, given the complexity of the migration phenomenon, future studies should account for local structural restrictions in the organization of care, the extension of a person’s network as a means of health care accessibility, diverse backgrounds and cultures, language barrier, health literacy, and the recent arrival status of migrants. This would allow researchers to understand the vast impact of social determinants of health on the wellbeing of a mother and newborn and consider irregular migrant women in health policies.

Investing in the prevention and protection of health during the first 1,000 days to improve the newborn’s outcomes of irregular migrant mothers should be prioritized to reduce adverse societal costs of maternal and reproductive lack of care. Adopting new evolving models of care will require great flexibility, yet this effort is an undeniable key factor for advancing and protecting both mothers and future generations.

### Supplementary Information

**Additional file 1.**

## Data Availability

The data that support the findings of this study are available from The Swiss Federal Statistical Office, but restrictions apply to the availability of these data, which were used under licence for the current study and are not publicly available. Data are, however, available from the corresponding author (CM) upon reasonable request and with permission of the Swiss Federal Statistical Office.

## Abbreviations

AOs: Adverse Outcomes
SGA: Small for Gestational Age
LBW: Low Birth Weight
VLBW: Very Low Birth Weight
PTB: Preterm Birth
EU: European Union
EFTA: European Free Trade Association
CH: Switzerland
SD: Standard Deviation
OECD: Organization for Economic Cooperation and Development
FSO: Federal Statistical Office
