# Supplementary material for: Are pregnancy outcomes affected by the lack of legal status? A demographic study based on 850,288 live births in Switzerland
Source: BMC Pregnancy Childbirth. 2023 Aug 5;23:567. doi: 10.1186/s12884-023-05870-5 (PMC10403826; doi:10.1186/s12884-023-05870-5)
Supplement: Supplementary file 1 — Additional file 1. [file 12884_2023_5870_MOESM1_ESM.docx]

Supplementary Materials

Table 1, Swiss 10th percentile for Gestational Age (Annex)

| Gestational age at birth  (weeks) | Mean weight  (gr) | Standard deviation (SD) | 10th percentile |
| --- | --- | --- | --- |
| 24 | 652 | 119.94 | 500 |
| 25 | 731 | 196.23 | 520 |
| 26 | 845 | 386.53 | 570 |
| 27 | 950 | 221.32 | 675 |
| 28 | 1135 | 460.32 | 770 |
| 29 | 1269 | 583.29 | 900 |
| 30 | 1479 | 540.72 | 1050 |
| 31 | 1586 | 354.26 | 1180 |
| 32 | 1781 | 355.70 | 1340 |
| 33 | 1990 | 393.47 | 1530 |
| 34 | 2224 | 404.41 | 1750 |
| 35 | 2466 | 432.63 | 1950 |
| 36 | 2698 | 430.05 | 2170 |
| 37 | 2952 | 445.62 | 2410 |
| 38 | 3214 | 424.36 | 2700 |
| 39 | 3361 | 409.22 | 2860 |
| 40 | 3495 | 415.46 | 2990 |
| 41 | 3605 | 414.25 | 3100 |
| 42 | 3689 | 419.93 | 3170 |
| 43 | 3687 | 764.27 | 3130 |

Table 2, 10th percentile for CH residents only

| Gestational age at birth  (weeks) | Mean weight  (gr) | Standard deviation (SD) | 10th percentile |
| --- | --- | --- | --- |
| 24 | 646 | 119.78 | 490 |
| 25 | 729 | 193.03 | 530 |
| 26 | 825 | 196.46 | 570 |
| 27 | 941 | 222.77 | 675 |
| 28 | 1114 | 379.46 | 770 |
| 29 | 1267 | 545.34 | 920 |
| 30 | 1466 | 524.85 | 1040 |
| 31 | 1575 | 327.13 | 1170 |
| 32 | 1781 | 354.52 | 1350 |
| 33 | 1989 | 405.91 | 1520 |
| 34 | 2214 | 412.95 | 1740 |
| 35 | 2455 | 441.04 | 1950 |
| 36 | 2690 | 422.08 | 2170 |
| 37 | 2942 | 441.16 | 2400 |
| 38 | 3202 | 419.92 | 2690 |
| 39 | 3355 | 406.09 | 2860 |
| 40 | 3491 | 413.01 | 2985 |
| 41 | 3602 | 413.13 | 3090 |
| 42 | 3695 | 424.51 | 3170 |
| 43 | 3657 | 461.86 | 3130 |

Table 3, Unadjusted logistic regressions between residency status and SGA, PTB, LBW, VLBW and any of these outcomes

|  | **SGA (n= 854,749)** | | | **PTB (n= 850,411)** | | | **LBW (n=993,856)** | | | **VLBW (n=993,856)** | | | **Any AO (n=850,288)** | | |
| --- | --- | --- | --- | --- | --- | --- | --- | --- | --- | --- | --- | --- | --- | --- | --- |
|  | OR | [95% CI] | p | OR | [95% CI] | p | OR | [95% CI] | p | OR | [95% CI] | p | OR | [95% CI] | p |
| **Residency** |  | | | | | | | | | | | | | | |
| Swiss resident | 1 (ref.) |  |  | 1 |  |  | 1 |  |  | 1 |  |  | 1 |  |  |
| Irregular migrant | .99 | .88-1.10 | 0.830 | 1.38 | 1.24-1.55 | 0.0 | 1.26 | 1.13-1.40 | 0.0 | 2.03 | 1.61-2.55 | 0.0 | 1.15 | 1.05-1.25 | 0.002 |
| Regular migrant | .93 | .92-.95 | 0.0 | .94 | .92-.96 | 0.0 | .93 | .92-.95 | 0.0 | 1.09 | 1.04-1.14 | 0.00 | .93 | .92-.95 | 0.0 |
|  | **SGA (n= 250,327)** | | | **PTB (n= 250,327)** | | | **LBW (n=255,307)** | | | **VLBW (n=255,307)** | | | **Any AO (n=250,279)** | | |
| **Migration status** |  | | | | | | | | | | | | | | |
| Regular | 1 (ref.) |  |  | 1 |  |  | 1 |  |  | 1 |  |  | 1 |  |  |
| Irregular | 1.06 | .95-1.18 | 0.321 | 1.47 | 1.31-1.64 | 0.0 | 1.39 | 1.24-1.56 | 0.0 | 1.92 | 1.50-2.45 | 0.0 | 1.23 | 1.13-1.34 | 0.0 |

AO, Adverse Outcome; SGA Small for gestational age; LBW, Low birth weight; VLBW, Very low birth weight; PTB, Preterm birth; EU, European Union; EFTA, European Free Trade Association; CH, Switzerland.

|  | SGA/SGACH (n=251,654) | | | PTB (n=251,654) | | | LBW (n=251,604) | | | VLBW (n=251,604) | | | Any AO (n=251,604) | | |
| --- | --- | --- | --- | --- | --- | --- | --- | --- | --- | --- | --- | --- | --- | --- | --- |
|  | aOR | [95% CI] | p | aOR | [95% CI] | p | aOR | [95% CI] | p | aOR | [95% CI] | p | aOR | [95% CI] | p |
| **Residency** |  | | | | | | | | | | | | | | |
| Regular migrant | 1 (ref.) |  |  | 1 |  |  | 1 |  |  | 1 |  |  | 1 |  |  |
| Irregular migrant | .81 | .72-.91 | 0.0 | 1.25 | 1.11-1.40 | 0 | 1.04 | .92-1.17 | 0.514 | 1.36 | 1.06-1.76 | 0.016 | .96 | .88-1.05 | 0.392 |
| Extra EU/EFTA | .70 | .56-.89 | 0.003 | 1.42 | 1.15-1.76 | 0.001 | 1.17 | .93-1.46 | 0.174 | 1.65 | 1.04-2.61 | 0.034 | .98 | .84-1.16 | 0.839 |
| Africa | 1.04 | .79-1.37 | 0.797 | 1.26 | .93-1.72 | 0.138 | 1.34 | 1.01-1.78 | 0.045 | 1.91 | 1.07-3.40 | 0.029 | 1.09 | .87-1.37 | 0.432 |
| Latin America | .67 | .54-.82 | 0 | 1.10 | .90-1.34 | 0.354 | .78 | .63-.96 | 0.017 | .90 | .56-1.44 | 0.655 | .84 | .72-.98 | 0.023 |
| Asia | .73 | .52-1.02 | 0.067 | 1.10 | 0.78-1.55 | 0.598 | .90 | .63-1.28 | 0.557 | 1.39 | .68-2.81 | 0.364 | .86 | .66-1.11 | 0.249 |
| Unknown | .97 | .89-1.06 | 0.455 | .82 | .73-.91 | 0 | .89 | .80-.99 | 0.033 | 1.03 | .81-1.33 | 0.793 | .92 | .86-.99 | 0.029 |
| **Parity** |  | | | | | | | | | | | | | | |
| One | 1 (ref.) |  |  | 1 |  |  | 1 |  |  | 1 |  |  | 1 |  |  |
| Two | .65 | .63-.67 | 0 | 1.03 | 1.00-1.07 | 0.060 | .92 | .89-.96 | 0 | .91 | .83-.99 | 0.037 | .77 | .75-.78 | 0 |
| Three | .59 | .57-.62 | 0 | 1.23 | 1.17-1.29 | 0.0 | 1.08 | 1.03-1.14 | 0.003 | 1.07 | .94-1.23 | 0.286 | .80 | .78-.83 | 0 |
| 4 + children | .62 | .57-.67 | 0 | 1.53 | 1.42-1.65 | 0.0 | 1.32 | 1.21-1.43 | 0 | 1.42 | 1.18-1.75 | 0.00 | .93 | .87-.98 | 0.012 |
| **Maternal civil status** |  | | | | | | | | | | | | | | |
| Married | 1(ref.) |  |  | 1 |  |  | 1 |  |  | 1 |  |  | 1 |  |  |
| Not married | 1.31 | 1.27-1.35 | 0 | 1.19 | 1.15-1.24 | 0.0 | 1.33 | 1.28-1.38 | 0 | 1.47 | 1.33-1.62 | 0 | 1.29 | 1.26-1.33 | 0 |
| **Canton of childbirth** |  | | | | | | | | | | | | | | |
| Other cantons | 1 (ref.) |  |  | 1 |  |  | 1 |  |  | 1 |  |  | 1 |  |  |
| Geneva | 1.07 | 1.02-1.13 | 0.005 | 1.38 | 1.31-1.46 | 0 | 1.51 | 1.44-1.60 | 0 | 1.97 | 1.72-2.26 | 0 | 1.20 | 1.15-1.25 | 0 |
| Vaud | 1.19 | 1.15-1.24 | 0 | 1.24 | 1.18-1.30 | 0 | 1.38 | 1.31-1.45 | 0 | 1.98 | 1.75-2.25 | 0 | 1.21 | 1.17-1.25 | 0 |
| Zurich | .93 | .90-.97 | 0 | 1.16 | 1.11-1.21 | 0 | 1.12 | 1.07-1.18 | 0 | 1.74 | 1.55-1.95 | 0 | 1.01 | .99-1.05 | 0.331 |
| Basel | 1.03 | .96-1.10 | 0.416 | 1.37 | 1.28-1.48 | 0 | 1.44 | 1.34-1.56 | 0 | 2.99 | 2.53-3.52 | 0 | 1.15 | 1.09-1.22 | 0 |
| Bern | .98 | .94-1.03 | 0.540 | 1.30 | 1.22-1.36 | 0 | 1.32 | 1.24-1.39 | 0 | 2.31 | 2.02-2.64 | 0 | 1.11 | 1.06-1.15 | 0 |

Table 4, Controlled logistic regressions between newborn outcomes and the independent social variables for regular vs. irregular migrants only

AO, Adverse Outcome; SGA Small for gestational age; LBW, Low birth weight; VLBW, Very low birth weight; PTB, Preterm birth; EU, European Union; EFTA, European Free Trade Association; CH, Switzerland

|  | SGA (n= 850,411) | | | PTB (n= 850,411) | | | LBW (n=854,699) | | | VLBW (n=854,699) | | | Any AO (n=850,288) | | |
| --- | --- | --- | --- | --- | --- | --- | --- | --- | --- | --- | --- | --- | --- | --- | --- |
|  | aOR | 95% CI | p | aOR | 95% CI | p | aOR | 95% CI | p | aOR | 95% CI | p | aOR | 95% CI | p |
| **Residency** |  | | | | | | | | | | | | | | |
| Swiss resident | 1 (ref.) |  |  | 1 |  |  | 1 |  |  | 1 |  |  | 1 |  |  |
| Irregular migrant | .76 | .68-.85 | 0.0 | 1.18 | 1.05-1.32 | 0.004 | .95 | .85-1.06 | 0.375 | 1.43 | 1.13-1.81 | 0.003 | .90 | .83-.99 | 0.022 |
| Regular migrant | .93 | .91-.94 | 0.0 | .93 | .92-.95 | 0 | .92 | .91-.94 | 0.0 | 1.08 | 1.04-1.14 | 0.00 | .92 | .91-.94 | 0.0 |
| **Period** | **ORs are reported for irregular migrants only** | | | | | | | | | | | | | | |
| 2005-2007 | .52 | .24-1.13 | .100 | .65 | .29-1.49 | .309 | .73 | .54-.97 | .029 | .88 | .46-1.73 | .729 | .55 | .31-.99 | .047 |
| 2008-2015 | .74 | .65-.85 | 000 | 1.13 | .99-1.30 | .071 | .96 | .83-1.10 | .524 | 1.53 | 1.15-2.03 | .004 | .89 | .80-.98 | .024 |
| 2016-2018 | .83 | .67-1.03 | .087 | 1.37 | 1.10-1.70 | .005 | 1.14 | .90-1.43 | .271 | 1.65 | .98-2.78 | .060 | .98 | .83-1.16 | .819 |

Table 5, Sensitivity analysis for three periods of time

AO, Adverse Outcome; SGA Small for gestational age; LBW, Low birth weight; VLBW, Very low birth weight; PTB, Preterm birth; EU, European Union; EFTA, European Free Trade Association; CH, Switzerland


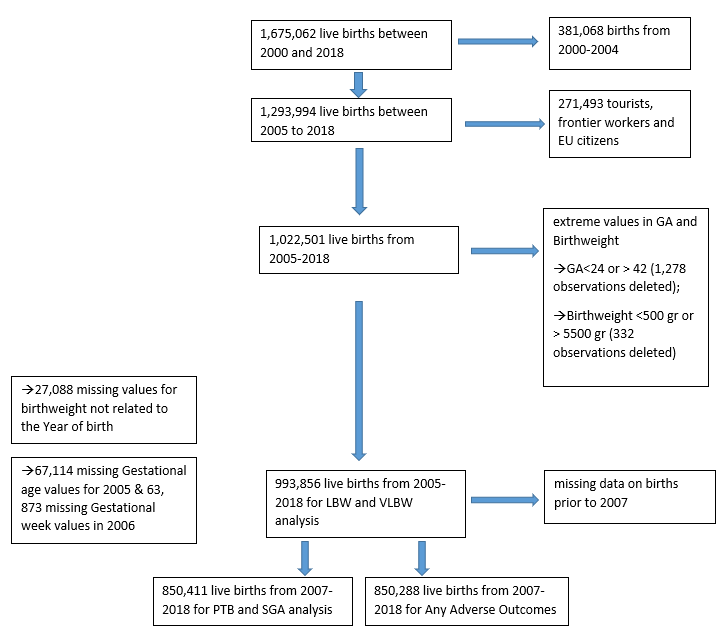


Figure 1, Data clearing flow-chart
